# Dietary intakes, nutritional and biochemical status of 6 months to 12-year-old children before the COVID-19 pandemic era: the South East Asian Nutrition Survey II Indonesia (SEANUTS II) study in Java and Sumatera Islands, Indonesia

**DOI:** 10.1017/S1368980024001654

**Published:** 2025-01-07

**Authors:** Aria Kekalih, Dian Novita Chandra, Listya Tresnanti Mirtha, Ilse Khouw, Gerard Wong, Rini Sekartini

**Affiliations:** 1 SEANUTS Indonesian Team/Faculty of Medicine, Universitas Indonesia, Depok, Indonesia; 2 Community Medicine Department, Faculty of Medicine, Universitas Indonesia, Depok, Indonesia; 3 Department of Nutrition, Faculty of Medicine, Universitas Indonesia, Depok, Indonesia; 4 FrieslandCampina, Stationsplein 4, Amersfoort, The Netherlands; 5 Department of Child Health, Faculty of Medicine, Universitas Indonesia, Depok, Indonesia

**Keywords:** Nutritional status, Child growth, Micronutrient deficiencies, Dietary intakes, Indonesian children

## Abstract

**Objective:**

The South East Asian Nutrition Survey II Indonesia aimed to provide up-to-date data on dietary intake, nutritional and biochemical status of children aged 0·5–12 years in Indonesia 2019–2020.

**Design:**

Multistage cluster sampling, stratified by geographical location.

**Setting:**

Out of forty-six targeted districts in Indonesia, the study only covered twenty-one districts/cities in Java and Sumatera islands, Indonesia due to COVID-19 pandemic.

**Participants:**

A total of 2475 children aged 0·5–12 years were included.

**Result:**

The growth (weight-for-age, height-for-age, weight-for-height and BMI-for-age) of Indonesian pre-school- and school-aged children was below the WHO standards. The prevalence of stunting in Java and Sumatera islands was 20·6 and 33·4 % in urban and rural areas, respectively. Stunting was higher in the 1·0–3·9-year age group, boys and rural areas. Overall, 9–12 percent of all children were overweight -obese, with 23·7 % of urban 7–12-year-olds having the highest prevalence.

Anaemia was 22·8 % in < 5-year-old and highest in < 1-year-old children. Fe, Zn, vitamins A and D insufficiency was observed in 20·3 %, 11·9 %, 1·9 % and 27·1 % of the children. Dietary intakes of energy, fibre, Ca, Fe, Zn, vitamins A, B_1_, C and vitamin D below the Indonesian RDA were prevalent and observed in more than half of the children.

**Conclusion:**

High stunting, increasing trends of overweight/ obesity, anaemia, serum vitamin D insufficiency, inadequate energy and micronutrient intake in children highlighted the triple burden of malnutrition in Java and Sumatera, Indonesia’s most populous regions in 2019–2020, shortly before COVID-19 pandemic era.

## Graphical abstract

**Figure:**
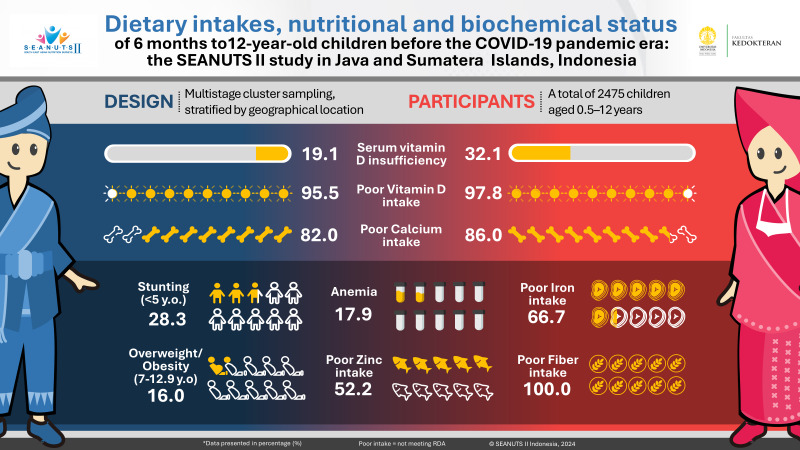

Malnutrition in children is a global major public health problem, especially in low- and middle-income countries (LMIC). Malnutrition threats include undernutrition like stunting (height-for-age, wasting (weight-for-height) and underweight (weight-for-age), as well as overnutrition like overweight and obesity (BMI-for age) and micronutrient deficiencies (e.g. Fe, vitamin-A and Zn)^(1)^. These multiple malnutrition problem address public health concern in many low- and middle-income country, known as ‘triple burden of malnutrition’^(2–4)^.

Undernutrition poses a serious threat to survival, health and performance during childhood and adulthood. The recent global estimates suggest that stunting affected 22% of under-5 children (149 million), while nearly 7% of children were wasted (45 million), and 13% were underweight (88 million)^(5)^. The World Health Organization statistics indicate that Southeast Asia and Africa have the highest burden of childhood undernutrition globally. Southeast Asia is the home of nearly two-fifth of all stunted children (36%), and half of all wasted (53%) and underweight children (50 %) in the world^(6,7)^.

According to the Global Burden of Disease, in 2009, malnutrition was the most important risk factor leading to mortality and disability^(8)^, while in 2019, it still ranked on the sixth place^(8)^. The 2018 Indonesian national survey, *Riset Kesehatan Dasar (Riskesdas*), showed that, among under-5 children, the prevalence of underweight was 17·7 %, stunting 30·8 %, and overweight and obesity combined 8·0 %. There was a decrease compared with the 2013 Riskesdas data, in which the prevalence of underweight, stunting, and overweight and obesity combined was 19·6 %, 37·2 %, and 11·9 %, respectively^(9,10)^. Although the incidence of malnutrition among Indonesian children has declined since 2013^(9–11)^, the current prevalence of undernutrition remains high compared with neighbouring countries, e.g. Malaysia and Thailand^(11)^.

To our knowledge, recent data on micronutrient status and risk factors associated with nutritional problems among Indonesian children before COVID-19 pandemic era are sparse. The most recent study on Indonesia micronutrient and nutritional status were the first SEANUTS study (2013) and the Indonesia Basic Health Survey (2018). Java-Sumatera are the most populated islands in Indonesia, with the most established health facilities and economic infrastructure, while also being the gateway to international transports which may escalate the risk of infectious disease transmission. Malnourished children are at higher risk of more severe impact of any infections, like diarrhoea, upper respiratory tract infection and especially COVID-19. Therefore, normal nutrition status and food consumption are critical to maintain their immunity. It is important to collect information on anthropometric nutritional status indices, biochemical parameters, dietary intakes and other factors associated with nutritional status to develop nutrition planning to address the triple burden of malnutrition.

This study was part of the South East Asian Nutritional Survey II (SEANUTS II), a multi-centric study simultaneously conducted among children aged 0·5–12 years in four countries: Indonesia, Malaysia, Thailand and Vietnam. The initial objective of SEANUTS II was to provide up-to-date data on nutritional status, food consumption and nutritional biochemical among children aged 0·5–12 years in whole Indonesia. However, due to the COVID-19 pandemic entering Indonesia in March 2020, the study coverage was limited to children in Java-Sumatera islands recruited between September 2019 and March 2020. The results of anthropometric variables, micronutrient status (Hb, ferritin, Zn, vitamins A, B_12_ and D) and the intakes of selected macronutrient and micronutrient will be discussed in this paper.

## Methods

### Study design and sampling procedure

This cross-sectional study was conducted in September 2019 to March 2020. The sample size estimation was based on stunting prevalence in Indonesia (37·2 % for children < 5 years and 30·7 % for children ≥ 5 years)^(9)^. The overall sample size of 7595 participants was calculated considering a 95 % confidence level, 10 % dropout rate, 2·0 design effect and 5 % margin of error for assessing children’s nutritional status by age group. More information on the SEANUTS II protocol can be found in a separate publication.

A multi-stage stratified random sampling was deployed in participant selection. From each selected regency (stage one), one district (stage two) was randomly selected, followed by the selection of one sub-district (stage three) and one hamlet (stage four). In stage five, children under 6 years were randomly selected from a list of households provided by the head of the hamlet (*rukun warga*), whereas the 7- to 12-year-old children were randomly identified from the elementary school list provided by the local government (sub-district or *kecamatan*). For families with multiple eligible siblings, only the youngest was selected. Children with physical disability, chronic illness, a history of hospitalization in the past 3 months, and feeling unwell during data collection was excluded.

However, due to the COVID-19 pandemic, the data were only collected in twenty-one out of forty-six selected regencies. Furthermore, only fifteen regencies (Java and Sumatera) met the minimum sample size to be extrapolated into the national estimation. In total, 2475 children aged 6 months to 12 years were included in the present analysis. However, the sample size was sufficient to maintain a precision margin of < 10 % and a power to compare output by gender and location that exceeds 80 %.

### Measurements

#### Anthropometry

Weight was measured in duplicate to the nearest 0·1 kg using SECA digital weighing scale 874 and 334. Infants and toddlers were measured on a flat and stable surface with the assistance of research staff. All children were weighed in minimal clothing, without shoes and jewelry or other ornaments. If the two measurements recorded > 0·1 kg difference, the measurements were repeated, and the median of three measurements was used as the final value. The scale was calibrated daily using a weight standard of 10 kg to assure the accuracy.

Height was also measured in duplicate to the nearest 0·1 cm using two approaches. First, we applied recumbent length for children < 2 years (SECA 417 infantometer). The children laid down with their head touching the fixed headboard, straight legs with their toes facing the sky. While the child’s mother helped to hold the child’s head in the vertical Frankfurt plane, the research staff aligned the infant’s legs and moved the foot piece to rest firmly on the infant’s heels. The third measurement was performed if the two measurements recorded > 0·5 cm difference. Second, we applied standing height for children aged ≥ 2 years using SECA 217 stadiometer. Children stood barefoot on a stadiometer platform without any hair ornaments, jewelry, buns or braids, and research staff ensured that the children stood with their hips, legs, heels and back straight and their hands on their sides.

BMI was calculated by dividing the measured weight (kg) by square of height (m).

#### Nutritional status

Nutritional status was classified using the WHO growth standards for 0–5 years (WHO 2006)^(12)^ and WHO growth reference for 5–19 years (WHO 2007)^(13)^. The z-scores for weight-for-age, height-for-age, BMI-for-age, and weight-for-height were determined using the software WHO Anthro version 3.1.0 (WHO, Geneva, Switzerland) for children aged ≤ 5 years; the WHO AnthroPlus version 1.0.3 (WHO, Geneva, Switzerland) was used for children aged > 5 years. Biologically implausible calculated z-scores were excluded from the analysis. The lower cut-off of –6 was used for height-for-age and weight-for-height and –5 for weight-for-age and BMI-for-age; the upper cut-off of 6 was used for height-for-age and 5 for other indices. The nutritional status was classified as stunting, wasting and underweight according to a cut-off point score < –2 sd for height-for-age, weight-for-height and weight-for-age, respectively. For children < 5 and ≥ 5 years old, BMI-for-age > 2 sd and > 1 sd was used to identify overweight, respectively, while > 3 sd or > 2 sd was used to classify obesity, respectively.

#### Biochemistry

Blood samples were obtained from 369 children (15 percent of total subjects) using a 95 percent confidence level, 10 % dropout rate, 20 % design effect and 7·5 % absolute precision for anemia by age group. A maximum of 14·5 ml early-morning whole venous blood samples were collected from each child by trained phlebotomists in EDTA tubes. Prior to this blood sample collection, a 12-hour overnight fast was requested for school-aged children (7–12·9 years old) only. The collected blood samples were kept in a standard storage box with an ice pack and transported immediately to an accredited laboratory for analysis or storage at –80°C. Standard methods of lab analysis were conducted, such as SLS-Hb for Hb assessment, chemiluminescent microparticle immunoassay (CMIA) for ferritin and vitamin B_12_, inductively coupled plasma MS (ICP-MS) for Zn, HPLC for vitamin A, direct competitive chemiluminescence immunoassay for vitamin D, nephelometry for alpha(1)-acid glycoprotein (AGP), and Immunoturbidimeter-CMIA for hsCRP assessment.

Anaemia was defined as Hb < 110 g/l for children < 5 years, < 115 g/l for 5–11 years, < 120 g/l for > 12 years^(14)^. Fe deficiency was defined as serum ferritin < 12 µg/l for children < 5 years and < 15 µg/l for ≥ 5 years, among those without inflammation (CRP ≤ 5 mg/l and AGP ≤ 1 g/l)^(15)^. In case of inflammation, ferritin level was adjusted by multiplying with a correction factor of 0·77 for incubation stage (CRP > 5 mg/l and AGP ≤ 1 g/l), 0·53 for early convalescence stage (CRP > 5 mg/l & AGP > 1 g/l), and 0·75 for late convalescence stage (CRP ≤ 5 mg/l & AGP > 1 g/l)^(16)^. Zn deficiency was defined as serum Zn < 65 µg/dl for children < 10 years (without fasting), < 70 µg/dl for ≥ 10 years females (with fasting), and < 74 µg/dl for ≥ 10 years males (with fasting)^(17)^. Vitamin A deficiency was classified as mild if the serum retinol was 0·35–0·7 µmol/l and as severe if retinol serum was < 0·35 µmol/l^(18)^. Vitamin B_12_ deficiency was defined as serum vitamin B_12_ < 150 pmol/l^(19)^. Vitamin D status was classified as insufficient if 25-hydroxyvitamin D was between 25–50 nmol/l and as deficient if the level was < 25 nmol/l^(20)^.

#### Dietary intake

A single multiple-pass 24-hours food recall was administered to assess energy, macronutrient and micronutrient intakes through a face-to-face interview with mothers or primary caregivers. Household measures and photographs of food portion sizes were used to improve recall accuracy.

Calculation of dietary intakes was performed using the NutriSurvey software for Windows 2007. Nutrient contents were derived mainly from the Indonesian Food Composition Tables where available^(21)^. Missing nutrients were adopted from several FCT, such as from the UK (McCance and Widdowson’s The Composition of Foods Integrated Dataset, 2021), Malaysia (https://myfcd.moh.gov.my/), Japan 2015, and USDA (https://fdc.nal.usda.gov/). Raw foods were transformed into cooked foods for single food items and composite dishes by calculating the yield and retention factors^(22)^. Following the calculation of energy expenditure with BMR and physical activity level assumption^(23)^ within 99 % CI, 100 subjects were excluded due to reporting implausible energy intakes^(24)^. Afterwards, individual dietary intakes were compared with the Indonesian Estimated Average Requirement and RDA^(25)^.

### Data collection

Data collection was conducted from September 2019 to March 2020. As the research staffs travelled throughout the country, all data were recorded on paper questionnaires and then later transferred into the electronic data capturing system VIEDOC. This study was conducted according to the guidelines laid down in the Declaration of Helsinki, and all procedures involving research study participants were approved by the Research Ethics Committee of Cipto Mangunkusumo Hospital and Faculty of Medicine Universitas Indonesia, registered as protocol no 19-10-0046 and approval letter no. 0031/UN2.F1/ETIK/2019. Written informed consent and assent was obtained from all subjects’ parents and subjects.

### Data analysis

The descriptive data values and differences between sex and residence (urban/ rural) were performed using the SPSS Complex Sampling procedure with weighting factors (SPSS version 20, IBM). In order to extrapolate the data to the total population on Java-Sumatera islands, the weighting factors were obtained from the Indonesia National Statistics Bureau based on age, sex and urban/rural residence per district using the 2010–2015 Population Census Report. Categorical data were analysed using *χ* ^2^ test. For numerical data, unpaired *t* test or the Mann–Whitney test was used to compare the difference between two groups. For comparison involving more than two groups, ANOVA or the Kruskal–Wallis test was used. In biochemical analysis, no weighted variables were used. A *P* value < 0·05 was considered to be statistically significant.

## Results

The results of SEANUTS II Indonesia have provided the latest data on nutritional status for representative samples of Indonesian children from Java and Sumatera. Table 1 shows the number of children who participated in the study as well as the estimated total population of children, by age group, sex and residence. Distribution of participating children based on sex ratio and residence (urban and rural) was similar to that in the population.

**Table 1:** Number and percentage of study subjects by age group, sex and residence

Age group	0·5–0·9 years			1·0–3·9 years			4·0–6·9 years			7·0–12·9 years			0·5–4·9 years			Total (0·5–12·9 years)		
	Sample		Estimated population	Sample		Estimated population	Sample		Estimated population	Sample		Estimated population	Sample		Estimated population	Sample		Estimated population
	*n*	%		*n*	%		*n*	%		*n*	%		*n*	%		*n*	%	
Urban																		
Boys	88	47·8	635 743	253	49·9	1 558 817	117	54·9	2 118 026	191	45·9	2 798 866	400	49·5	2 897 973	649	49·2	7 111 452
Girls	96	52·2	648 678	254	50·1	1 448 8797	96	45·1	1 177 028	225	54·1	3 689 059	408	50·5	2 627 439	671	50·8	6 963 562
Total	184	100·0	1 284 421	507	100·0	3 007 614	213	100·0	3 295 054	416	100·0	6 487 925	808	100·0	5 525 412	1320	100·0	14 075 014
Rural																		
Boys	67	54·5	821 955	254	51·8	2 823 365	94	52·8	2 390 172	160	44·0	4 894 275	374	53·2	4 563 465	575	49·8	10 929 767
Girls	56	45·5	534 343	236	48·2	2 752 646	84	47·2	1 918 406	204	56·0	5 982 295	329	46·8	3 858 618	580	50·2	11 187 690
Total	123	100·0	1 356 298	490	100·0	5 576 011	178	100·0	4 308 578	364	100·0	10 876 570	703	100·0	8 422 083	1155	100·0	22 117 457
Urban and rural																		
Boys	155	50·5	1 457 698	507	50·9	4 382 182	211	54·0	4 508 197	351	45·0	7 693 142	774	51·2	7 461 438	1224	49·5	18 041 219
Girls	152	49·5	1 183 021	490	49·1	4 201 443	180	46·0	3 095 434	429	55·0	9 671 354	737	48·8	6 486 057	1251	50·5	18 151 253
Total	307	100·0	2 640 719	997	100·0	8 583 625	391	100·0	7 603 631	780	100·0	17 364 496	1511	100·0	13 947495	2475	100·0	36 192 472

### Anthropometry and nutritional status

Online Supplementary Table 1 shows the means and standard errors for weight, height and BMI of the children by age group, sex and residence. Overall, boys were heavier and taller in the age groups 0·5–0·9 and 4–6·9 years old while this was the case for girls in the oldest age group. Significant differences in height between urban and rural were commonly found in boys and total children (boys and girls), especially among those aged 1·0–3·9 and 7·0–12·9 years.

**Fig. 1:**
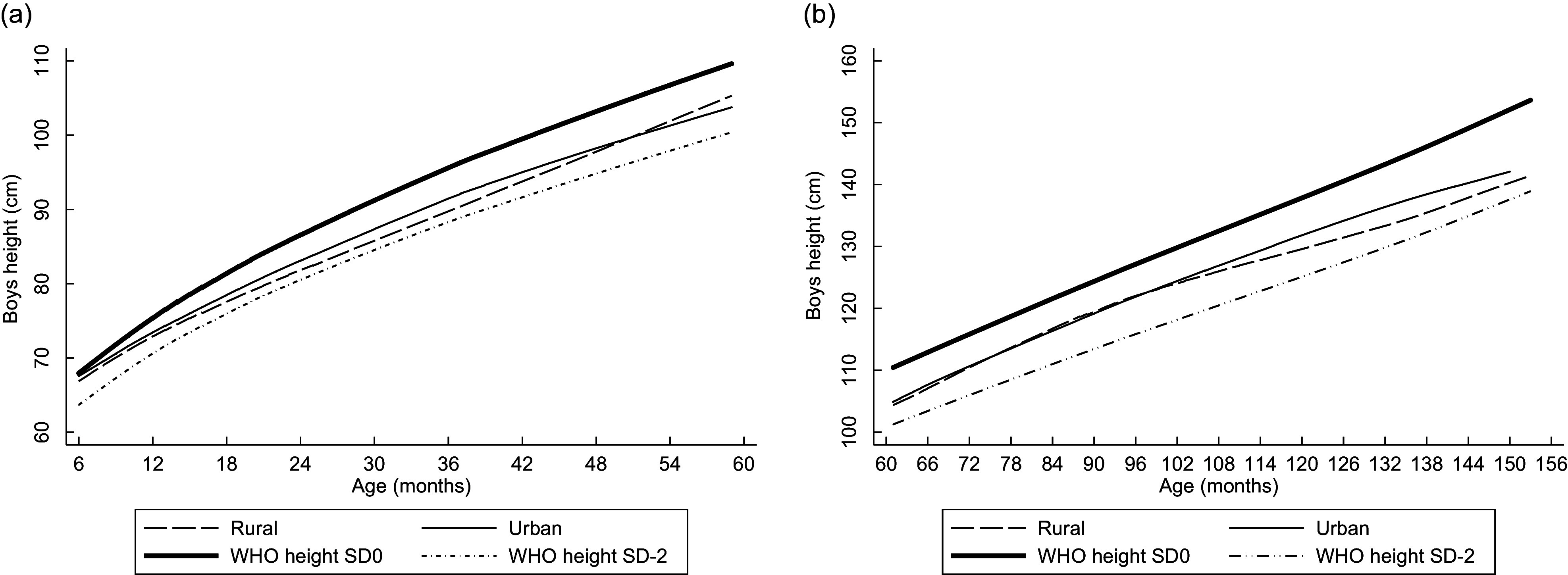
Linear graph: Anthropometric nutritional status indices by age group, sex and residence. (a) Mean height of boys aged 0·5–4·9 years. (b) Mean height of boys aged 5–12·9 years.

**Fig. 2:**
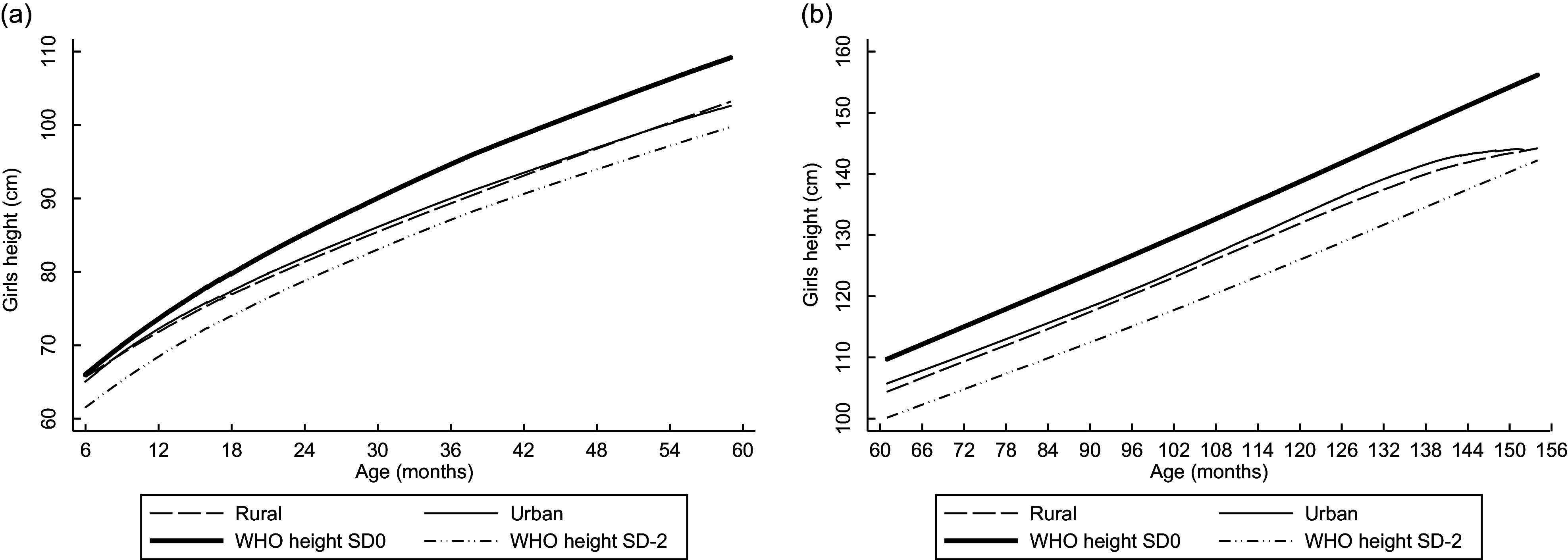
Linear graph: Anthropometric nutritional status indices by age group, sex and residence. (a) Mean height of girls aged 0·5–4·9 years. (b) Mean height of girls aged 5–12·9 years.

**Fig. 3:**
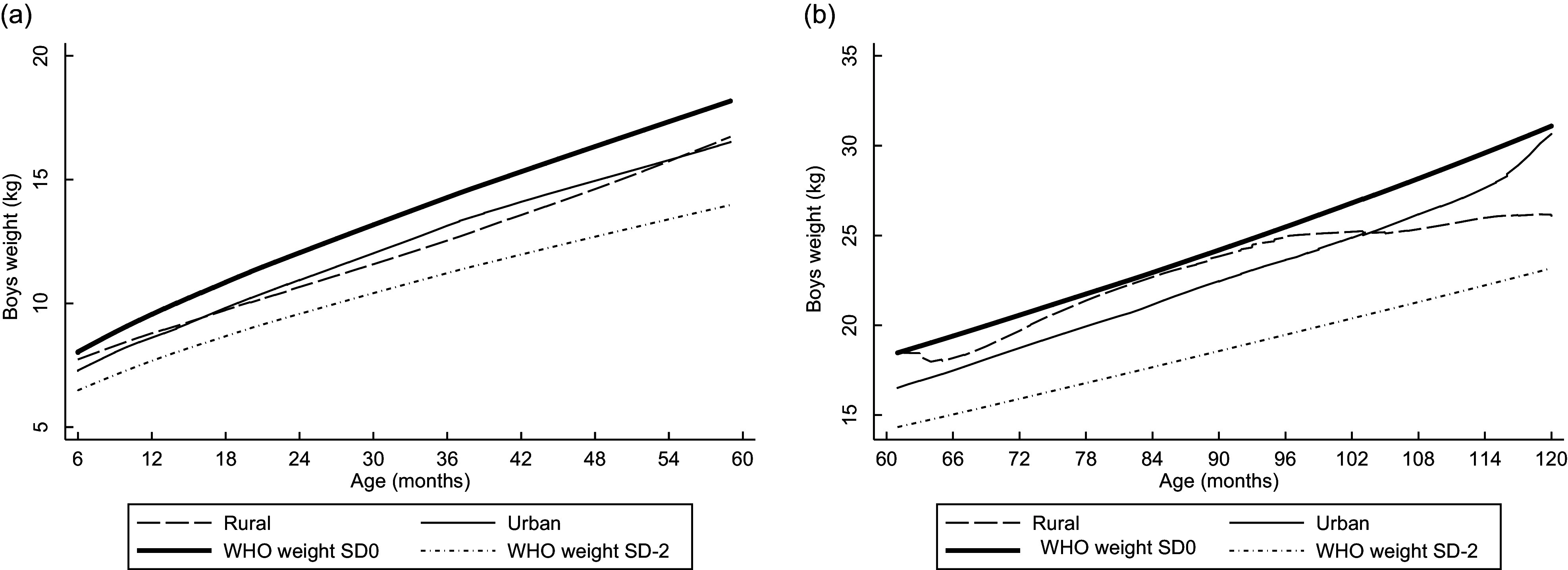
Linear graph: Anthropometric nutritional status indices by age group, sex and residence. (a) Mean weight of boys aged 0·5–4·9 years. (b) Mean weight of boys aged 5·0–9·9 years.

**Fig. 4:**
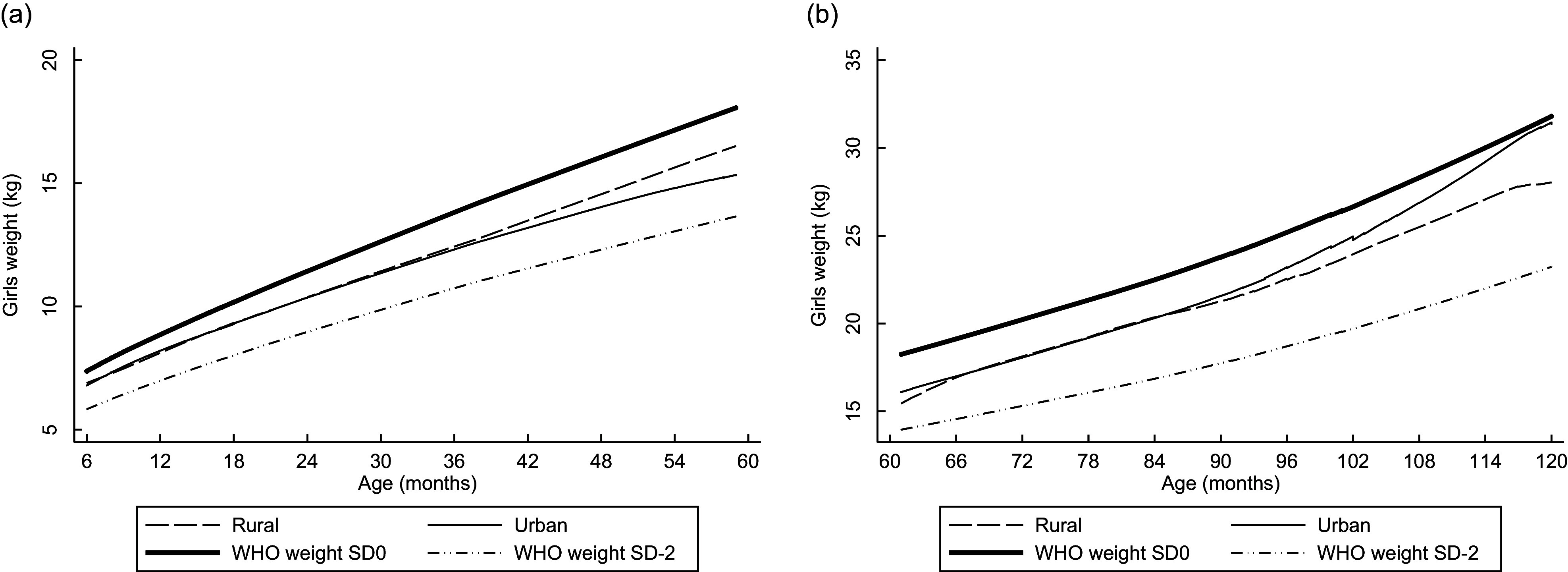
Linear graph: Anthropometric nutritional status indices by age group, sex and residence. (a) Mean weight of girls aged 0·5–4·9 years. (b) Mean weight of girls aged 5·0–9·9 years.

Linear plots of height and weight per age compared with the WHO standards are shown in Figs 1–4. Beginning on the curve at 6 months of age, the mean height of the children was close to the WHO standard (sd 0). In contrast, the disparity between boys’ and girls’ mean height according to the WHO standard sd 0 widened as children aged and approached sd –2 at the end of 12 years old. The weights of rural girls lagged behind those of their urban counterparts, whereas boys’ weight patterns fluctuated, and urban boys’ curves increased with age more rapidly than those of rural boys.

Table 2 shows the prevalence of malnutrition for the different age groups, sex and residence. The overall stunting prevalence of under 5-year-old children in Java and Sumatera is 20·6% and 33·4% in urban and rural areas, respectively. Stunting in rural children was significantly higher than in urban children. The prevalence of stunting was highest in the 1·0–3·9-year age group especially in boys and rural areas (46·2%). In rural areas, the prevalence of stunting was consistently above 20% in all age groups. Wasting was prevalent in 5·8% and 3·3% of the under 5-year-old children in urban and rural areas, respectively. The prevalence of underweight in rural (19·1%) was similar to that in urban areas (18·9 %). In urban areas, underweight was more prevalent in boys than girls especially in the younger age groups. The highest prevalence of overweight (15·3%) and obesity (8·4%) was observed among urban children aged 7·0–12·9 years, whereas the prevalence of overweight plus obesity for rural children for the same age group was 12·0%.

**Table 2:**
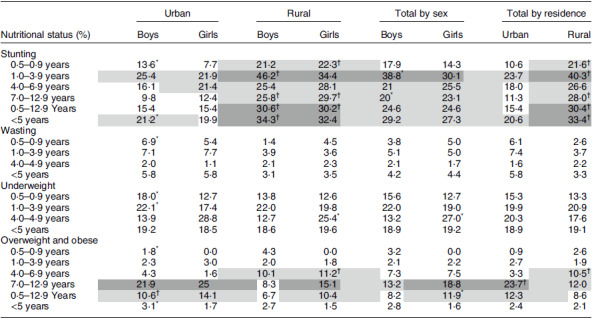
Nutritional status by age group, sex and residence

Light grey shading indicates high (stunting: 20–< 30 %, wasting and overweight: 10–<15 %); dark grey very high prevalence (stunting: ≥ 30 %, wasting and overweight: ≥ 15 %) public health threshold^(49)^.

*Prevalence (%) was at the highest and signiﬁcantly different between the sexes (*P* < 0·05).

†Prevalence (%) was at the highest and signiﬁcantly different between the urban and rural areas (*P* < 0·05).

### Biochemical status

Results on the prevalence of anaemia, Fe deficiency and Zn deficiency is shown in Table 3a. Overall, anaemia in boys was 17·3 % and did not differ with girls at 18·4 %. Anaemia was more prevalent in urban than rural areas in children aged 4·0–6·9 years (21·6 % *v*. 3·4 % resp.). Among total children (urban and rural), the prevalence of anaemia was higher in the younger age groups (0·5–0·9 years: 48·6 %, 1·0–3·9 years: 20·5 %) than that in the older age groups (4·0–6·9 years: 13·6 %, 7·0–12·9 years: 7·5 %). Among children aged 7·0–12·9 years, inflammation-adjusted Fe deficiency was significantly higher in girls (14·8 %) than boys (3·4 %). Fe deficiency anaemia was higher in < 1-year-old children, and especially rural children and girls. Zn deficiency was higher in urban (17·4 %) compared with rural (5·7 %) irrespective of sex.

**Table 3a:** Prevalence (%) of anaemia, iron deficiency, and iron deficiency anaemia and zinc deficiency by age group, sex and residence

Nutrient problems	Age categories	Urban			Rural			Urban and Rural		
		Boys	Girls	Total	Boys	Girls	Total	Boys	Girls	Total
Anaemia	0·5–0·9 years	63·6	35·7	48·0	66·7	33·3	50·0	64·7	35·0	48·6
	1·0–3·9 years	24·1	17·5	20·3	17·1	23·8	20·8	20·3	20·7	20·5
	4·0–6·9 years	15·8	27·8	21·6^†^	0·0	6·7	3·4	9·1	18·2	13·6
	7·0–12·9 years	3·2	9·1	6·3	7·1	10·7	8·9	5·1	9·8	7·5
	0·5–12·9 years	20·0	19·0	19·5	14·5	17·6	16·1	17·3	18·4	17·9
Fe deficiency corrected for inflammation	0·5–0·9 years	27·3	21·4	24·0	50·0	16·7	33·3	35·3	20·0	27·0
	1·0–3·9 years	34·5	20·0	26·1	28·6	35·7	32·5	31·3	28·0	29·5
	4·0–6·9 years	21·1	16·7	18·9	14·3	13·3	13·8	18·2	15·2	16·7
	7·0–12·9 years	0·0	12·1^*^	6·3	7·1	17·9	12·5	3·4	14·8^*^	9·2
	0·5–12·9 years	18·9	17·1	17·9	20·5	25·3	23·0	19·7	20·9	20·3
Fe deficiency anaemia	0·5–0·9 years	18·2	7·1	12·0	50·0	16·7	33·3	29·4	10·0	18·9
	1·0–3·9 years	17·2	15·0	15·9	17·1	16·7	16·9	17·2	15·9	16·4
	4·0–6·9 years	10·5	11·1	10·8	0·0	6·7	3·4	6·1	9·1	7·6
	7·0–12·9 years	0·0	6·1	3·1	3·6	7·1	5·4	1·7	6·6	4·2
	0·5–12·9 years	10·0	10·5	10·3	12·0	12·1	12·1	11·0	11·2	11·1
Zn deficiency	0·5–0·9 years	27·3	35·7	32	16·7	16·7	16·7	23·5	30·0	27·0
	1·0–3·9 years	17·2	22·5^†^	20·3^†^	5·7	2·4	3·9	10·9	12·2	11·6
	4·0–6·9 years	21·1	33·3	27	14·3	6·7	10·3	18·2	21·2	19·7
	7·0–12·9 years	3·2	3·0	3·1	7·1	0·0	3·6	5·1	1·6	3·3
	0·5–12·9 years	14·4	20·0^†^	17·4^†^	8·4	3·3	5·7	11·6	12·2	11·9

Number of samples by age groups, 0·5–0·9 years (*n* 37), 1·0–3·9 years (*n* 146), 4·0–6·9 years (*n* 66), 7·0–12·9 years (*n* 120), 0·5–12·9 years (*n* 369).

* Prevalence (%) was at the highest and signiﬁcantly different between the sexes (*P* < 0·05).

† Prevalence (%) was at the highest and signiﬁcantly different between the urban and rural areas (*P* < 0·05).

Table 3b shows the prevalence of vitamin A deficiency, vitamin B_12_ deficiency and vitamin D insufficiency. Mild vitamin A deficiency was more prevalent in urban areas (3·6 %) compared with 0 % in rural areas. Vitamin B_12_ deficiency was low at 1·6 % in all children. The prevalence of vitamin D insufficiency was significantly higher in girls than boys (32·1 % *v*. 19·1 %) for 0·5- to 12·9- year-old groups and at highest in 7- to 12·9-year-old children (57·4 % *v*. 28·8 % for girls *v*. boys). Profile of micronutrient deficiencies for age groups of 0·5–4·9 years old is shown in online Supplementary Table 2.

**Table 3b:** Prevalence (%) of vitamin A deficiency (mild), vitamin B_12_ deficiency, vitamin D insufficiency and deficiency by age group, sex and residence

Nutrient problem	Age categories	Urban			Rural			Urban and& Rural		
		Boys	Girls	Total	Boys	Girls	Total	Boys	Girls	Total
Vit A deficiency (mild)	0·5–0·9 years	18·2	14·3	16	0·0	0·0	0·0	11·8	10·0	10·8
	1·0–3·9 years	0·0	2·5	1·4	0·0	0·0	0·0	0·0	1·2	0·7
	4·0–6·9 years	0·0	5·6	2·7	0·0	0·0	0·0	0·0	3·0	1·5
	7·0–12·9 years	0·0	3·0	1·6	0·0	0·0	0·0	0·0	1·6	0·8
	0·5–12·9 years	2·2	4·8^†^	3·6^†^	0·0	0·0	0·0	1·2	2·6	1·9
Vit B_12_ deficiency	0·5–0·9 years	0·0	7·1	4·0	0·0	0·0	0·0	0·0	5·0	2·7
	1·0–3·9 years	3·4	0·0	1·4	2·9	7·1	5·2	3·1	3·7	3·4
	4·0–6·9 years	0·0	0·0	0·0	0·0	0·0	0·0	0·0	0·0	0·0
	7·0–12·9 years	0·0	0·0	0·0	0·0	0·0	0·0	0·0	0·0	0·0
	0·5–12·9 years	1·1	1·0	1·0	1·2	3·3	2·3	1·2	2·0	1·6
Vit D insufficiency	0·5–0·9 years	9·1	7·1	8·0	0·0	16·7	8·3	5·9	10·0	8·1
	1·0–3·9 years	17·2	15	15·9	8·6	26·2^*^	18·2	12·5	20·7	17·1
	4·0–6·9 years	26·3	27·8	27·0	14·3	26·7	20·7	21·2	27·3	24·2
	7·0–12·9 years	25·8	54·5^*^	40·6	32·1	60·7^*^	46·4	28·8	57·4^*^	43·3
	0·5–12·9 years	21·1	28·6	25·1	16·9	36·3^*^	27·0	19·1	32·1^*^	26·0
Vit D deficiency	0·5–0·9 years	0·0	0·0	0·0	0·0	0·0	0·0	0·0	0·0	0·0
	1·0–3·9 years	0·0	0·0	0·0	00.	0·0	0·0	0·0	0·0	0·0
	4·0–6·9 years	0·0	0·0	0·0	0·0	0·0	0·0	0·0	0·0	0·0
	7·0–12·9 years	3·2	3·0	3·1	0·0	7·1	3·6	1·7	4·9	3·3
	0·5–12·9 years	1·1	1·0	1·0	0·0	2·2	1·1	0·6	1·5	1·1

Number of samples by age groups, 0·5–0·9 years (*n* 37), 1·0–3·9 years (*n* 146), 4·0–6·9 years (*n* 66), 7·0–12·9 years (*n* 120), 0·5–12·9 years (*n* 369).

* Prevalence (%) was at the highest and signiﬁcantly different between the sexes (*P* < 0·05).

† Prevalence (%) was at the highest and signiﬁcantly different between the urban and rural areas (*P* < 0·05).

### Dietary intake

While average intake of macro- and micronutrients is summarised in online Supplementary Table 3, Table 4 reveals that more than 50 % of all children consumed energy, fibre, Ca, Fe, Zn, and vitamins A, B_1_, C and D did not meet Indonesian RDA. In 0·5- to 12·9-year-old children, more rural children were not meeting recommended intakes for protein, vit B_2_, and C compared to urban children. Nutrient intake not meeting RDA was more prevalent in the older children in both rural and urban areas. For example, in < 1-year-old children, nutrients with > 50 % children intake not meeting RDA were fiber, Fe, vit B_12_, C and D. But then at the oldest age groups (7–12·9 years old) prevalent low intakes were found in energy, Ca, Zn, vitamin A and vitamin B_1_.

**Table 4:** Percentage of children consuming selected macronutrients and micronutrients and not meeting the Indonesian RDA by age group, sex and residence

Variables	Urban			Rural			Urban and Rural		
	Boys	Girls	Total	Boys	Girls	Total	Boys	Girls	Total
0·5–0·9 years									
Energy	55·4	37·7	46·1	51·7	44·7	48·9	53·2	41·0	47·6
Protein	30·8	42·9^*^	37·2	33·9	36·3	34·9	32·7	39·8^*^	35·9
Total dietary fibre	100·0	100·0	100·0	100·0	100·0	100·0	100·0	100·0	100·0
Ca	26·2^*^	9·4	17·4	15·7	22·7^†^	18·5	19·9	15·7	18·0
Fe	83·1	90·0^*^	86·7	75·5	92·3	82·2	78·5	91·1	84·2
Zn	13·3^*^	7·8	10·4	3·8	16·3	8·7	7·5	11·8^*^	9·5
Vitamin A	7·8	17·8^*,†^	13·1	4·9	9·8	6·8	6·0	14·0^*^	9·7
Thiamine B_1_	64·2^*,†^	43·8	53·5	41·6	53·4	46·2	50·6	48·3	49·6
Riboflavin B_2_	44·0	36·8	40·2	30·3	46·0	36·5	35·8	41·1	38·2
Cobalamin	72·5^†^	78·8	75·8^†^	52·1	70·0	59·2	60·2	74·7	66·8
Vitamin C	41·8	55·7	49·1	49·0	58·8	52·9	46·1	57·2^*^	51·1
Vitamin D	89·3	97·2^*^	93·5	97·3	99·1	98·0	94·1	98·1^*^	95·9
1·0–3·9 years									
Energy	58·3^*^	51·5	55·0	67·0	60·5	63·8	63·9^*^	57·4	60·7
Protein	19·7^*^	15·4	17·6	31·2^†^	31·4^†^	31·3^†^	27·2	25·9	26·5
Total dietary fibre	100·0	100·0	100·0	100·0	100·0	100·0	100·0	100·0	100·0
Ca	57·8	68·3^*^	62·9	76·0^†^	77·0	76·5	69·6	74·0^*^	71·8
Fe	42·2	59·7	50·7	66·0^†^	60·1	63·1	57·6	60·0	58·8
Zn	13·5	15·8^*^	14·6	26·9^†^	28·9^†^	27·9^†^	22·2	24·3	23·2
Vitamin A	32·3	37·9^*^	35·0	47·7	49·8^†^	48·7^†^	42·3	45·7	44·0
Thiamine B_1_	30·5	34·3^*^	32·4	51·9^†^	41·4	46·7^†^	44·4	38·9	41·7
Riboflavin B_2_	19·3	24·7	21·9	27·9^†^	23·8	25·9	24·9	24·1	24·5
Cobalamin	24·3^*^	23·4	23·9	44·9^†^	38·1	41·6^†^	37·7	33·0	35·4
Vitamin C	45·2	49·7	47·3	69·2	76·2^†^	72·7	60·7	67·1	63·8
Vitamin D	91·0	94·3	92·6	92·6	98·6	95·6	92·0	97·1^*^	94·5
4·0–6·9 years									
Energy	56·9	66·0^*^	60·1	65·2	82·5	72·7	61·3	76·2^*^	67·3
Protein	21·3	16·2	19·5	26·3	32·0	28·8	24·0	26·0	24·8
Total dietary fibre	100·0	100·0	100·0	99·7	100·0	99·9	99·9	100·0	99·9
Ca	79·1	93·1^*^	84·1	91·7	88·2	90·2	85·9	90·1	87·5
Fe	75·9	73·9	75·2	67·6	77·4	71·8	71·4	76·1	73·3
Zn	54·8	56·9	55·5	46·3	61·7	53·0	50·2	59·9	54·1
Vitamin A	49·0	69·8^*^	56·4	50·4	43·1	47·2	49·8	53·3	51·2
Thiamine B_1_	36·5	40·3	37·8	38·5	37·5	38·0	37·5	38·6	38·0
Riboflavin B_2_	26·6	21·4	24·7	24·9	14·7	20·5	25·7^*^	17·3	22·3
Cobalamin	21·0	19·5	20·4	20·6	20·0	20·3	20·8	19·8	20·4
Vitamin C	71·0	64·8	68·8	78·7	79·6	79·1	75·2	74·0	74·7
Vitamin D	89·8	97·4^*^	92·5	96·1	92·6	94·6	93·2	94·5	93·7
7·0–12·9 years									
Energy	83·3^*^	80·6	81·8	89·6	84·4	86·7	87·3	83·0	84·9
Protein	61·5	57·1	59·0	65·4	75·5^†^	70·9^†^	64·0	68·5	66·5
Total dietary fibre	100·0	100·0	100·0	100·0	100·0	100·0	100·0	100·0	100·0
Ca	97·3	95·1	96·0	97·6	99·9^*,†^	98·9^†^	97·5	98·1	97·8
Fe	62·1	64·0	63·2	65·1	67·0	66·1	64·0	65·8	65·0
Zn	63·2	66·3	64·9	76·8^†^	73·9	75·2^†^	71·8	71·0	71·4
Vitamin A	78·1	76·3	77·1	84·5	87·1^†^	85·9^†^	82·2	83·0	82·6
Thiamine B_1_	83·4	79·2	81·0	89·7^†^	88·8^†^	89·2^†^	87·4	85·2	86·1
Riboflavin B_2_	77·8^*^	63·9	69·9	80·5^*^	71·0	75·3	79·5^*^	68·3	73·3
Cobalamin	57·7	58·0	57·9	66·6	66·3	66·4	63·4	63·2	63·2
Vitamin C	91·9^*^	77·9	83·9	86·4	88·5^†^	87·6	88·4	84·5	86·2
Vitamin D	97·7	98·2	98·0	99·9^*,†^	99·7	99·8^†^	99·1	99·1	99·1
0·5–12·9 years									
Energy	67·8	68·4	68·1	75·6	76·5	76·0	72·6	73·4	73·0
Protein	38·0	40·4	39·2	45·7	55·7^†^	50·8^†^	42·7	49·9^*^	46·3
Total dietary fibre	100·0	100·0	100·0	99·9	100·0	100·0	100·0	100·0	100·0
Ca	77·7	81·7^*^	79·7	84·7	88·7	86·7	82·0	86·0^*^	84·0
Fe	63·6	67·1^*^	65·3	66·7	68·3	67·5	65·5	67·8	66·7
Zn	46·0	49·1	47·6	52·0	58·2	55·1	49·7	54·8	52·2
Vitamin A	54·0	62·1^*^	58·0	61·7	67·0	64·4	58·7	65·2	61·9
Thiamine B_1_	56·4	60·3^*^	58·3	65·2	67·1	66·1	61·7	64·5	63·1
Riboflavin B_2_	47·2	46·3	46·7	51·1^†^	49·0	50·1^†^	49·6	48·0	48·8
Cobalamin	40·6	46·2^*^	43·4	49·8^†^	51·9	50·9^†^	46·2	49·8	48·0
Vitamin C	71·6^*^	67·9	69·8	77·6	82·6^†^	80·1	75·2	77·0	76·1
Vitamin D	93·2	97·2^*^	95·2	97·0	98·2	97·6	95·5	97·8^*^	96·7

* Prevalence (%) was at the highest and signiﬁcantly different between the sexes (*P* < 0·05).

† Prevalence (%) was at the highest and signiﬁcantly different between the urban and rural areas (*P* < 0·05).

## Discussion

### Statement of principal findings

SEANUTS II Indonesia has shown that stunting children in Indonesia, specifically Java and Sumatera, can be indicated as a high public health problem due to a consistent prevalence of > 20 % across all age groups. The average of height and weight of children from Java and Sumatera remain below the WHO growth, primarily in rural areas. Overall, overweight-obese was highest in urban 7- to 12-year-old children. Blood biochemistry analysis showed that anaemia was mostly prevalent in the younger children as was Fe deficiency. In contrast, vitamin D insufficiency was mainly an issue in the older children. Nutrient intake data showed that most children did not meet recommended intake for energy, fiber, vitamin D, Ca and Fe.

Stunting remains a serious nutritional problem in Java-Sumatera, the most populated islands in Indonesia even though the prevalence of stunting decreased over the past years. Stunting for children < 5 years decreased from 34·3 % in 2011 (SEANUTS I)^(26)^ to 30·8 % in 2018 (Basic Health Survey/Riskesdas 2018)^(10)^, 27·7 % in 2019 (SSGBI) and 24·4 % in 2021 (SSGI)^(27)^. At the same time, the ambition of the Indonesian government is to reduce stunting prevalence to below 14 % by 2024^(27)^.

Stunting for under 5-year-olds is more prevalent in rural areas compared with urban, which is comparable to some other studies^(28–32)^. Continued lack of access to health care, limited infrastructure (i.e. prolonged travel time health care facilities) and poor hygiene and sanitation practices may partly explain the differences in the prevalence of stunting between rural and urban^(10,32)^. Other factors related to stunting are dietary habits and the required knowledge of parents and caregivers to provide healthy meals to their children, as revealed by Mulyaningsih et al. Stunting is a multifactorial public health issue and interventions to reduce stunting should cover multiple levels: not only focused on the child but also on family and community characteristics as well^(33)^.

On the other hand, overweight and obesity also requires attention especially in urban areas and in the older age groups. SEANUTS II Indonesia showed a prevalence of 23·7 % for urban children in the age of 7–12·9 years old compared with 12·0 % for rural children (in total 16 %). Possible contributors to developing overweight/ obesity are the eating habits of Indonesian children, with a high preference for high energy items such as fried foods and sugary drinks, together with insufficient physical activity, primarily in urban area^(34)^. Future analysis will focus on the associations of the children’s physical activity, their diet and their nutritional status.

Overweight and obesity is a growing issue in other Asian countries as well. SEANUTS II Malaysia, Thailand and Vietnam demonstrated even higher prevalence of overweight plus obesity of more than 30 % for the older children^(35)^. Moreover, in SEANUTS I, the prevalence of overweight plus obesity was between 5 % and 10 % for rural and urban 5- to 12·9-year-old children, respectively^(26)^, showing an increase of overnutrition over approximately 10 years’ time. It is imperative to implement effective policies and programmes to prevent further increase of the overweight/ obesity prevalence in Indonesia.

From perspective of blood biochemistry findings, SEANUTS II highlighted important figures of micronutrient insufficiency like anaemia, Zn and vitamin D. Anaemia was shown to be mostly prevalent in the youngest age groups (48·6 %) and the children of 1–3·9 years old (20·5 %), while the prevalence is lower in the older children (13·6 % and 7·5 % in the children 4–6·9 and 7–12·9 years old, respectively). Similar results were found in the other SEANUTS II countries and in SEANUTS I Indonesia data as well. A possible explanation of the high prevalence of anaemia in the youngest children could be the low quality of complementary feeding. Late introduction of animal protein source in complementary feeding also stated in previous study as the common factor risk of anaemia in 0·5- to 0·9-year-old children^(28,36)^. Sunardi et al.^(37)^ showed that inadequate Zn intake was linked to anaemia. However, SEANUTS II only showed 9·5 % and 23·2 % of the children aged 0·5–0·9 and 1–3·9 years, respectively, to have inadequate Zn consumption. At the same time, Sunardi et al. showed that children not consuming infant and young child formula had 8·6 times higher risk of having anemia. Possible explanation is that infant and young child formula is fortified with important micronutrients, including Fe to support child growth and development.

Zn deficiency was lower in the school aged children compared with the younger children. This is in contrast with a study by Awasthi et al. in India, which showed that zn deficiency is higher in 6- to –11-year-old children compared with younger children^(38)^. Children food preference in school aged children in Indonesia might contributed to this findings, as they lack of animal protein, fruit and vegetables for Zn source^(39)^. Further analysis in the SEANUTS II data will need to be done to confirm these micronutrients deficiencies findings and their factor risks.

In contrast to anaemia, which is less prevalent in older children, vitamin D insufficiency was more prevalent in older children. Similar trends were shown a systematic review by Oktaria et al, who found that vitamin D insufficiency and deficiency is common Southeast Asian children with newborns and females most at risk^(40)^. However, the trends in age groups were different, specifically the Oktaria study found that insufficiency was at the highest among newborns (90 %), lowest among 6 months old (0·9 %), and around 40 % for 2–12 years old. The risk in older groups also stressed by study in Indonesia capital city by Pulungan et al. showed that 37·5 % of 7- to 12-year-old children had vitamin D insufficiency, while 1·7 % were vitamin D deficient^(41)^. Clothing style, outdoors activity and milk consumption were amongst possible factor risks. More analyses are required for better insights into the factors leading to vitamin D insufficiency and deficiency and how to treat and prevent this public health issue.

SEANUTS II Indonesia found a prevalence of 1·9 % of mild vitamin A deficiency, which was higher in urban compared with rural (3·6 % *v*. 0·0 % resp.). These findings are lower than the previous SEANUTS study in 2011^(26)^. Indonesia also implemented WHO recommendation of vitamin A supplementation with a dose of 100·000 IU in infants aged 6–11 months and 200·000 IU twice a year in young children aged 12–59 months. These findings might be related to vitamin A supplementation coverage, as correlates to the mother’s education, economic level and social media exposure^(42)^.

SEANUTS II Indonesia showed the following nutrients to be a main concern regarding sufficient intakes: energy, fibre, Ca and vitamin D. There are differences between age groups. For example, Fe intake in the children 0·5–0·9 years old was an issue, while Ca was not. At the same time, a high percentage of school-aged children failed to meet the recommended intake for some additional nutrients, including Zn, vitamins A, B_1_, B_12_ and C. This finding was consistent with the results of a study conducted by Sunardi et al., which revealed that > 50 % of Indonesian children between 6 and 36 months old had inadequate intake of energy, Ca and vitamin D. Moreover, their study found that intake of fat and folate was also less than optimal^(37)^.

A possible reason for the high percentage of young children not meeting the recommendation for nutrient intakes could be the quality of complementary feeding (CF). Rizki and Rahayu (2020)^(43)^ and Hijra et al. (2016)^(44)^ showed that inappropriate CF practice was profound in East Java and Central Sulawesi, which could affect the prevalence of stunting in 1- to 3year-old children. Majority of infants in Indonesia are introduced too early to CF compared to the WHO recommendation (2021)^(45)^. Moreover, diets are characterised by a low variety and low frequency of CF and consist mainly of staple foods with poor nutritional quality, such as rice, cereals or noodles. Protein-rich foods of animal origins are introduced too late in the diets of children, while the consumption of fruit and vegetables, especially during the early CF period, is poor. In addition, late introduction of meats could be linked to the high prevalence of anaemia in this age group^(46)^. In contrast, a significant proportion of both urban and rural children in Indonesia are consuming energy-dense/nutrient-poor snacks (e.g. fritters) and sugary drinks (e.g. sweetened condensed milk or iced sweetened tea) during the CF period^(47)^. Further analysis within the SEANUTS II data could confirm these findings.

SEANUTS II Indonesia showed that the older the children, the higher the percentages of not meeting recommended levels for nutrient intakes. Similar trends were already observed in SEANUTS I Indonesia^(26)^. However, there are limited studies focusing on nutrient intakes in Indonesian school children. More studies should help in providing a better understanding of the status of nutrient intakes in this population including the health effects linked to inadequate intakes.

Note that this study’s findings were just prior to the outbreak of COVID-19 in Indonesia. With the halting of social and economic progress in Indonesia, because of COVID-19 epidemic, the triple burden of malnutrition problem is anticipated to rise in the near future. Therefore, existing education programmes on balanced diets should be strengthened, particularly in most affected areas, to prevent the growing trends of malnutrition

### Strengths and limitations

The present study has several strengths. SEANUTS II Indonesia explored the nutritional status in children between 6 months to 12 years old in Java and Sumatera with a wide range of assessments. Blood biochemistry, dietary intake and physical activity and fitness (not reported here) in particular are complementary to other national nutrition surveys conducted in Indonesia. As applied in all four countries of SEANUTS II study, the assessments of children’s anthropometry, food consumption, nutritional status and biochemical status were conducted using a standardised protocol. In Indonesia, as we assigned three teams for data collection simultaneously, we ensured the internal validity and reliability of the measurements by SEANUTS ID nutritionist experts. Furthermore, all research staffs received standardised trainings, including training for anthropometry measurements, data collection using electronic data capturing, outlier data verification and food intake measurements using the WHO criteria^(12)^.

However, there are some limitations as well. First, the COVID-19 pandemic has prevented us to collect data at the national level, so SEANUTS II Indonesia is only representative for Java and Sumatera islands. Second, the biochemical analysis only involved 15 % of total subject chosen purposively which might deteriorate the representativeness toward population especially when comparing subgroups. Therefore, biochemistry data are not reported as weighted. This article is only limited for reporting the descriptive results and comparison between age groups, sex and residence. Association analysis to investigate the risk factors of malnutrition will be discussed in a subsequent publication.

### Implications for research, policy and practice

The first 1000 d of a child’s life have become a target for nutrition and health programs in Indonesia, particularly in the 260 districts and ten provinces with the highest rates of stunting^(31)^. Health policies focusing on children’s nutritional status should be consistent and continuous. This needs collaborative efforts between health sector and other stakeholders, for example socio-economic or infrastructure development in rural areas to minimise the gap between urban and rural areas, even in the most populated and well-resourced like Java and Sumatera.

To accelerate the raise of awareness of stunting and micronutrients deficiency in children, penta helix approach that involves academia, business, government, community and mass media is needed. An example of the involvement of the private sector is the community nutrition empowerment program as part of the corporate social responsibility and food fortification program in food products for children^(48)^. Vigorous socialisation through mass media also highly increased the awareness towards importance of young children nutrition.

### Conclusions

High stunting, increasing rates of overweight/ obesity, anaemia, serum vitamin D insufficiency and inadequate energy and micronutrient intake in children signified the triple burden of malnutrition in Indonesia’s densely populated islands of Java and Sumatera in 2019–2020, just right before COVID-19 pandemic era. The COVID-19 pandemic has the potential to escalate the burden of malnutrition, which necessitates a national, regional or global collaboration effort to avert it.

## Supporting information

Kekalih et al. supplementary material Kekalih et al. supplementary material

## Supplementary material

For supplementary material accompanying this paper visit https://doi.org/10.1017/S1368980024001654

## Authors’ note

The SEANUTS II Indonesia Study Group comprises of the following individuals: Universitas Indonesia: Rini Sekartini, Dian Novita Chandra, Aria Kekalih, Listya Tresnanti, Dina Indah, Ari Prayogo, Roselyne Anggraini, Luthfi Saiful Arif, Wahyu Budi Santoso, Saptawati Bardosono, Aryono Hendarto, Soedjatmiko. FrieslandCampina: Ilse Khouw, Swee Ai Ng, Ye Sun, Panam Parikh, Yi Xiu Ng, Gerard Wong, Friska Navisa Ratri (FrieslandCampina Indonesia).
