# Supplementary material for: Dietary intakes, nutritional and biochemical status of 6 months to 12-year-old children before the COVID-19 pandemic era: the South East Asian Nutrition Survey II Indonesia (SEANUTS II) study in Java and Sumatera Islands, Indonesia
Source: Public Health Nutr. 2025 Jan 7;28(1):e1. doi: 10.1017/S1368980024001654 (PMC11736649; doi:10.1017/S1368980024001654)
Supplement: Kekalih et al. supplementary material [file S1368980024001654sup001.docx]

### Supplementary Table 1. Anthropometric characteristics of the children by age group, sex, and residence

| **Variables** | **Urban** | | | | | | **Rural** | | | | | | **Urban & Rural** | | | | | |
| --- | --- | --- | --- | --- | --- | --- | --- | --- | --- | --- | --- | --- | --- | --- | --- | --- | --- | --- |
|  | **Boys** | | **Girls** | | **Boys & Girls** | | **Boys** | | **Girls** | | **Boys & Girls** | | **Boys** | | **Girls** | | **Boys & Girls** | |
|  | **Mean** | **SE** | **Mean** | **SE** | **Mean** | **SE** | **Mean** | **SE** | **Mean** | **SE** | **Mean** | **SE** | **Mean** | **SE** | **Mean** | **SE** | **Mean** | **SE** |
| **0.5-0.9 years** |  |  |  |  |  |  |  |  |  |  |  |  |  |  |  |  |  |  |
| Weight (kg) | 8.0* | 0.11 | 7.4 | 0.09 | 7.6 | 0.08 | 8.0* | 0.14 | 7.3 | 0.11 | 7.7 | 0.10 | 8.0* | 0.09 | 7.3 | 0.07 | 7.7 | 0.06 |
| Height (cm) | 69.9*† | 0.34 | 68.1 | 0.33 | 69.0† | 0.25 | 68.7* | 0.37 | 67.2 | 0.46 | 68.1 | 0.30 | 69.3* | 0.26 | 67.7 | 0.27 | 68.6 | 0.19 |
| BMI (kg/m2) | 16.2 | 0.14 | 15.8 | 0.14 | 16.0 | 0.10 | 16.9*† | 0.19 | 16.1 | 0.18 | 16.6† | 0.14 | 16.6* | 0.12 | 15.9 | 0.11 | 16.3 | 0.08 |
| **1.0-3.9 years** |  |  |  |  |  |  |  |  |  |  |  |  |  |  |  |  |  |  |
| Weight (kg) | 11.6* | 0.16 | 11.2 | 0.14 | 11.4 | 0.11 | 11.4 | 0.14 | 11.3 | 0.16 | 11.3 | 0.10 | 11.5 | 0.10 | 11.3 | 0.11 | 11.4 | 0.07 |
| Height (cm) | 86.4† | 0.52 | 85.6 | 0.47 | 86.0† | 0.35 | 84.6 | 0.49 | 84.6 | 0.48 | 84.6 | 0.34 | 85.3 | 0.36 | 84.9 | 0.33 | 85.1 | 0.24 |
| BMI (kg/m2) | 15.5* | 0.09 | 15.2 | 0.09 | 15.3 | 0.07 | 15.8† | 0.08 | 15.7† | 0.12 | 15.7† | 0.07 | 15.7 | 0.06 | 15.5 | 0.08 | 15.6 | 0.05 |
| **4.0-6.9 years** |  |  |  |  |  |  |  |  |  |  |  |  |  |  |  |  |  |  |
| Weight (kg) | 17.7*† | 0.31 | 15.9 | 0.26 | 17.1 | 0.22 | 16.8 | 0.28 | 16.6 | 0.42 | 16.7 | 0.24 | 17.2* | 0.21 | 16.3 | 0.26 | 16.8 | 0.16 |
| Height (cm) | 107.3*† | 0.65 | 104.3 | 0.67 | 106.2 | 0.48 | 105.3 | 0.56 | 104.7 | 0.73 | 105.0 | 0.45 | 106.2* | 0.43 | 104.5 | 0.50 | 105.5 | 0.33 |
| BMI (kg/m2) | 15.3* | 0.15 | 14.5 | 0.12 | 15.0 | 0.11 | 15.0 | 0.16 | 15.1 | 0.30 | 15.1 | 0.16 | 15.2 | 0.11 | 14.9 | 0.17 | 15.0 | 0.10 |
| **7.0-12.9 years** |  |  |  |  |  |  |  |  |  |  |  |  |  |  |  |  |  |  |
| Weight (kg) | 28.9† | 0.54 | 31.8*† | 0.72 | 30.6† | 0.47 | 26.8 | 0.45 | 29.6* | 0.74 | 28.3 | 0.46 | 27.6 | 0.35 | 30.4* | 0.52 | 29.2 | 0.33 |
| Height (cm) | 131.5 | 0.68 | 133.1 | 0.78 | 132.4† | 0.53 | 129.6 | 0.73 | 131.1 | 0.81 | 130.4 | 0.56 | 130.3 | 0.50 | 131.8* | 0.56 | 131.2 | 0.38 |
| BMI (kg/m2) | 16.5† | 0.21 | 17.5*† | 0.26 | 17.1† | 0.17 | 15.9 | 0.20 | 16.7* | 0.25 | 16.3 | 0.17 | 16.1 | 0.14 | 17.0* | 0.18 | 16.6 | 0.12 |

* Mean value was at the highest and signiﬁcantly different between the sexes (P<0.05).

† Mean value was at the highest and signiﬁcantly different between the urban and rural areas (P<0.05).

### Supplementary Table 2. Prevalence (%) of micronutrient deficiencies for children 0.5-4.9 years old per sex, and residence and prevalence (%) of children 0.5-4.9 years old not meeting local RDA, per sex and residence

| **Nutrient problems** | **Urban** | | | **Rural** | | | **Urban & Rural** | | |
| --- | --- | --- | --- | --- | --- | --- | --- | --- | --- |
|  | **Boys** | **Girls** | **Total** | **Boys** | **Girls** | **Total** | **Boys** | **Girls** | **Total** |
| **Prevalence of micronutrients deficiencies** | | | | | | | | | |
| Anemia | 30.6 | 20.0 | 24.6 | 20 | 21.8 | 21.0 | 25.3 | 20.8 | 22.8 |
| Iron deficiency corrected for inflammation | 32.7 | 20 | 25.4 | 28 | 29.1 | 28.6 | 30.3 | 24.2 | 26.9 |
| Zinc deficiency | 22.4† | 26.2† | 24.6† | 8.0 | 5.5 | 6.7 | 15.2 | 16.7 | 16.0 |
| Vit A deficiency (mild) | 4.1 | 4.6 | 4.4† | 0.0 | 0.0 | 0.0 | 2.0 | 2.5 | 2.3 |
| Vit B12 deficiency | 2.0 | 1.5 | 1.8 | 2.0 | 5.5 | 3.8 | 2.0 | 3.3 | 2.7 |
| Vit D insufficiency | 18.4 | 18.5 | 18.4 | 8.0* | 23.6 | 16.2 | 13.1 | 20.8 | 17.4 |
| **Children consuming selected macronutrients and micronutrients below the local RDA** | | | | | | | | | |
| Energy | 54.5 | 51.4 | 53.0 | 63.6 | 61.0† | 62.4 | 60.2* | 57.1 | 58.7 |
| Protein | 20.2 | 23.9 | 22.0 | 30.9† | 31.0† | 31.0† | 26.9 | 28.1 | 27.5 |
| Fiber | 100.0 | 100.0 | 100.0 | 100.0 | 100.0 | 100.0 | 100.0 | 100.0 | 100.0 |
| Ca | 56.9 | 60.0 | 58.4 | 66.7 | 70.0* | 68.2 | 63.0 | 66.0 | 64.4 |
| Fe | 57.3 | 69.8* | 63.4 | 68.9 | 65.5 | 67.3 | 64.5 | 67.2 | 65.8 |
| Zn | 22.5 | 25.0 | 23.7 | 26.4 | 31.1 | 28.5 | 24.9 | 28.7* | 26.7 |
| Vit A | 30.0 | 39.5* | 34.6 | 39.4 | 41.7 | 40.4 | 35.8 | 40.8 | 38.2 |
| Vit B1 | 36.1 | 40.5 | 38.2 | 46.4 | 43.2 | 44.9 | 42.5 | 42.1 | 42.3 |
| Vit B2 | 22.5 | 29.0 | 25.7 | 27.3 | 25.5 | 26.5 | 25.5 | 26.9 | 26.2 |
| Vit B12 | 32.1 | 37.1 | 34.5 | 40.9 | 40.4 | 40.7 | 37.6 | 39.1 | 38.3 |
| Vit C | 52.6 | 54.3 | 53.4 | 64.6 | 71.4 | 67.7 | 60.1 | 64.5 | 62.1 |

* Prevalence (%) was at the highest and signiﬁcantly different between the sexes (P<0.05).

† Prevalence (%) was at the highest and signiﬁcantly different between the urban and rural areas (P<0.05).

**Supplementary Table 3. Intakes of selected macronutrients and micronutrients by age group, sex, and residence**

|  | **Urban** | | | | **Rural** | | | | **Urban & Rural** | | | |
| --- | --- | --- | --- | --- | --- | --- | --- | --- | --- | --- | --- | --- |
| **Variables** | **Boys** | | **Girls** | | **Boys** | | **Girls** | | **Boys** | | **Girls** | |
|  | **Mean** | **SE** | **Mean** | **SE** | **Mean** | **SE** | **Mean** | **SE** | **Mean** | **SE** | **Mean** | **SE** |
| **0.5-0.9 years** |  |  |  |  |  |  |  |  |  |  |  |  |
| Energy (kJ) | 707.2 | 20.71 | 695.8 | 18.61 | 699.8 | 21.04 | 680.7 | 25.85 | 702.7 | 14.59 | 688.7 | 15.26 |
| CHO (g) | 95.2 | 3.1 | 94.3 | 2.75 | 93.5 | 3.16 | 87.1 | 3.61 | 94.2 | 2.19 | 90.9 | 2.21 |
| Protein (g) | 18.7 | 0.7 | 18.8 | 0.67 | 18.3 | 0.69 | 18.2 | 0.9 | 18.5 | 0.49 | 18.5 | 0.54 |
| Fat (g) | 27.3 | 0.85 | 26.4 | 0.84 | 27.3 | 0.87 | 28.3 | 1.06 | 27.3 | 0.6 | 27.3 | 0.66 |
| Fiber (g) | 2.7 | 0.22 | 2.8† | 0.2 | 2.7 | 0.26 | 2.1 | 0.2 | 2.7 | 0.17 | 2.5 | 0.14 |
| Ca (mg) | 401.2 | 18.65 | 421.4 | 16.38 | 415.2 | 18.34 | 415.8 | 24.02 | 409.6 | 12.92 | 418.8 | 13.81 |
| Fe (mg) | 5.9 | 0.41 | 5.8 | 0.35 | 7.4† | 0.54 | 6.0 | 0.47 | 6.8* | 0.34 | 5.9 | 0.28 |
| Zn (mg) | 4.6 | 0.19 | 4.4 | 0.14 | 5.0 | 0.22 | 4.5 | 0.21 | 4.8 | 0.15 | 4.5 | 0.12 |
| Vit A (RAE) | 722.5* | 45.55 | 597.8 | 25.05 | 657.9* | 36.97 | 548.8 | 19.89 | 683.7* | 28.84 | 574.8 | 16.79 |
| Vit B1 (mg) | 0.3 | 0.03 | 0.4 | 0.03 | 0.4*† | 0.03 | 0.3 | 0.03 | 0.4 | 0.02 | 0.4 | 0.02 |
| Vit B2 (mg) | 0.7 | 0.05 | 0.6 | 0.04 | 0.7 | 0.05 | 0.6 | 0.06 | 0.7 | 0.04 | 0.6 | 0.03 |
| Vit B12 (ug) | 1.5 | 0.24 | 1.1 | 0.1 | 1.8* | 0.18 | 1.2 | 0.14 | 1.7* | 0.15 | 1.1 | 0.08 |
| Vit C (mg) | 62.4 | 3.74 | 59.9 | 3.69 | 65.4 | 5.35 | 57.1 | 4.81 | 64.2 | 3.27 | 58.6 | 2.94 |
| Vit D (ug) | 3.6 | 0.39 | 3.8 | 0.32 | 3.5 | 0.38 | 3.4 | 0.41 | 3.5 | 0.27 | 3.6 | 0.26 |
| **1.0-3.9 years** |  |  |  |  |  |  |  |  |  |  |  |  |
| Energy (kJ) | 1075.2† | 27.83 | 1014.2 | 20.76 | 961.5 | 24.33 | 969.7 | 24.57 | 1001.7 | 18.29 | 985.0 | 16.42 |
| CHO (g) | 139.0*† | 3.96 | 128.3 | 2.85 | 125.0 | 3.41 | 124.8 | 3.27 | 129.9 | 2.57 | 126.0 | 2.2 |
| Protein (g) | 35.3† | 1.01 | 33.0† | 0.8 | 28.0 | 0.8 | 27.9 | 0.82 | 30.6 | 0.64 | 29.7 | 0.58 |
| Fat (g) | 42.1 | 1.26 | 41.1 | 1.07 | 38.8 | 1.14 | 40.1 | 1.19 | 40.0 | 0.84 | 40.4 | 0.81 |
| Fiber (g) | 4.5 | 0.19 | 4.2 | 0.18 | 4.1 | 0.18 | 3.9 | 0.18 | 4.3 | 0.13 | 4.0 | 0.13 |
| Ca (mg) | 621.0*† | 24.48 | 529.9 | 19.71 | 507.3 | 24.41 | 486.7 | 19.98 | 547.5* | 17.46 | 501.5 | 14.03 |
| Fe (mg) | 8.8*† | 0.37 | 7.0 | 0.23 | 6.7 | 0.25 | 6.5 | 0.22 | 7.4* | 0.22 | 6.7 | 0.16 |
| Zn (mg) | 6.3*† | 0.25 | 5.2† | 0.16 | 4.6 | 0.18 | 4.7 | 0.17 | 5.2 | 0.15 | 4.9 | 0.12 |
| Vit A (RAE) | 636.6 | 29.34 | 562.8 | 25.4 | 568.9 | 33.82 | 558.8 | 33.81 | 592.9 | 22.91 | 560.2 | 21.87 |
| Vit B1 (mg) | 0.7 | 0.03 | 0.7 | 0.03 | 0.7 | 0.04 | 0.7 | 0.03 | 0.7 | 0.03 | 0.7 | 0.02 |
| Vit B2 (mg) | 1.0* | 0.04 | 0.9 | 0.03 | 0.9 | 0.04 | 0.9 | 0.03 | 1.0* | 0.03 | 0.9 | 0.02 |
| Vit B12 (ug) | 2.9 | 0.15 | 2.8† | 0.15 | 2.6 | 0.16 | 2.4 | 0.12 | 2.7 | 0.11 | 2.5 | 0.09 |
| Vit C (mg) | 56.5† | 2.88 | 51.2† | 2.78 | 36.1 | 2.63 | 30.8 | 2.32 | 43.3* | 1.97 | 37.8 | 1.81 |
| Vit D (ug) | 7.5*† | 0.47 | 5.9† | 0.34 | 4.8 | 0.33 | 4.5 | 0.28 | 5.7* | 0.28 | 5.0 | 0.22 |
| **4.0-6.9 years** |  |  |  |  |  |  |  |  |  |  |  |  |
| Energy (kJ) | 1372.8* | 51.44 | 1201.8† | 44.31 | 1285.8* | 48.29 | 1073.2 | 39.01 | 1325.9* | 35.29 | 1122.3 | 29.35 |
| CHO (g) | 175.7* | 7.45 | 148.6† | 5.48 | 161.0* | 6.16 | 131.9 | 4.91 | 167.8* | 4.85 | 138.3 | 3.67 |
| Protein (g) | 42.0 | 1.79 | 40.3† | 1.87 | 40.1* | 2.07 | 33.2 | 1.54 | 40.9* | 1.36 | 35.9 | 1.21 |
| Fat (g) | 55.6 | 2.12 | 49.5 | 2.31 | 53.4* | 2.43 | 46.4 | 1.89 | 54.4* | 1.6 | 47.6 | 1.46 |
| Fiber (g) | 4.8 | 0.35 | 4.7† | 0.3 | 5.1* | 0.36 | 3.7 | 0.25 | 4.9* | 0.25 | 4.1 | 0.2 |
| Ca (mg) | 600.8* | 42.66 | 480.2 | 37.26 | 504.6 | 37.28 | 504.4 | 47.1 | 549.0 | 28.46 | 495.2 | 30.42 |
| Fe (mg) | 8.0 | 0.47 | 8.2 | 0.41 | 9.6† | 0.61 | 8.1 | 0.56 | 8.9 | 0.38 | 8.2 | 0.35 |
| Zn (mg) | 6.1 | 0.36 | 5.5 | 0.29 | 5.9 | 0.32 | 5.3 | 0.4 | 6.0 | 0.24 | 5.4 | 0.25 |
| Vit A (RAE) | 894.2* | 125.67 | 459.7 | 45.31 | 977.2 | 170.76 | 664.5† | 64.02 | 938.9* | 105.16 | 586.3 | 40.75 |
| Vit B1 (mg) | 1.0* | 0.09 | 0.8 | 0.05 | 0.9 | 0.06 | 0.9 | 0.06 | 1.0 | 0.05 | 0.8 | 0.04 |
| Vit B2 (mg) | 1.3 | 0.1 | 1.0 | 0.05 | 1.4 | 0.11 | 1.2 | 0.07 | 1.3* | 0.08 | 1.1 | 0.04 |
| Vit B12 (ug) | 3.9 | 0.3 | 4.1 | 0.42 | 5.2* | 0.77 | 3.2 | 0.28 | 4.6* | 0.41 | 3.6 | 0.24 |
| Vit C (mg) | 35.9† | 4.36 | 39.0† | 5.12 | 24.0 | 2.6 | 24.9 | 3.72 | 29.5 | 2.58 | 30.3 | 3.1 |
| Vit D (ug) | 5.2 | 0.48 | 4.5 | 0.43 | 4.2 | 0.43 | 5.6 | 0.66 | 4.7 | 0.32 | 5.2 | 0.41 |
| **7.0-12.9 years** |  |  |  |  |  |  |  |  |  |  |  |  |
| Energy (kJ) | 1417.8† | 46.9 | 1419.5† | 32.68 | 1370.4 | 37.66 | 1281.5 | 31.16 | 1387.7 | 29.13 | 1333.7 | 22.6 |
| CHO (g) | 174.7 | 6.2 | 178.3† | 4.24 | 170.0* | 4.9 | 154.1 | 3.9 | 171.7* | 3.82 | 163.3 | 2.9 |
| Protein (g) | 45.9† | 1.46 | 46.2† | 1.27 | 39.4 | 1.22 | 37.3 | 1.07 | 41.8 | 0.94 | 40.7 | 0.84 |
| Fat (g) | 58.9 | 2.26 | 57.5 | 1.66 | 58.6 | 1.88 | 56.4 | 1.63 | 58.7 | 1.43 | 56.8 | 1.16 |
| Fiber (g) | 5.4 | 0.3 | 5.3 | 0.24 | 5.7 | 0.28 | 6.6*† | 0.34 | 5.6 | 0.2 | 6.1 | 0.22 |
| Ca (mg) | 397.3 | 19.87 | 430.4† | 28.51 | 346.0 | 18.96 | 338.2 | 14.58 | 364.7 | 13.58 | 373.1 | 15.09 |
| Fe (mg) | 8.6 | 0.31 | 8.5 | 0.35 | 7.9 | 0.29 | 7.7 | 0.25 | 8.1 | 0.21 | 8.0 | 0.21 |
| Zn (mg) | 5.9† | 0.21 | 6.1† | 0.29 | 5.3 | 0.17 | 5.0 | 0.15 | 5.5 | 0.13 | 5.4 | 0.15 |
| Vit A (RAE) | 396.7 | 30.95 | 485.8† | 33.39 | 363.2 | 40.3 | 314.9 | 24.0 | 375.4 | 25.74 | 379.5 | 20.18 |
| Vit B1 (mg) | 0.6 | 0.03 | 0.7† | 0.04 | 0.5 | 0.04 | 0.6 | 0.03 | 0.6 | 0.02 | 0.6 | 0.03 |
| Vit B2 (mg) | 0.8 | 0.04 | 0.9† | 0.04 | 0.8 | 0.04 | 0.8 | 0.03 | 0.8 | 0.03 | 0.9 | 0.03 |
| Vit B12 (ug) | 2.7 | 0.16 | 3.0† | 0.16 | 2.6 | 0.18 | 2.5 | 0.16 | 2.6 | 0.12 | 2.7 | 0.11 |
| Vit C (mg) | 21.6 | 2.13 | 33.2*† | 3.48 | 20.8 | 3.41 | 19.0 | 1.75 | 21.1 | 2.07 | 24.3 | 1.85 |
| Vit D (ug) | 3.0† | 0.27 | 3.4† | 0.28 | 1.6 | 0.14 | 1.7 | 0.15 | 2.1 | 0.15 | 2.4 | 0.15 |
| **0.5-12.9 years** |  |  |  |  |  |  |  |  |  |  |  |  |
| Energy (kJ) | 1274.8 | 23.56 | 1235.6 | 19.16 | 1198.5* | 20.56 | 1142.8 | 18.42 | 1228.2* | 15.4 | 1178.1 | 13.21 |
| CHO (g) | 161.1† | 3.17 | 155.6† | 2.44 | 151.0* | 2.63 | 140.1 | 2.28 | 154.9 | 2.02 | 146.0 | 1.66 |
| Protein (g) | 40.3† | 0.78 | 40.0† | 0.74 | 35.1 | 0.71 | 33.4 | 0.63 | 37.1 | 0.53 | 36.0 | 0.48 |
| Fat (g) | 51.8 | 1.08 | 50.0 | 0.94 | 50.1 | 1.01 | 49.4 | 0.94 | 50.8 | 0.73 | 49.7 | 0.66 |
| Fiber (g) | 4.8 | 0.15 | 4.8 | 0.13 | 4.9 | 0.14 | 5.3† | 0.18 | 4.9 | 0.1 | 5.1 | 0.11 |
| Ca (mg) | 507.0*† | 14.85 | 458.4† | 14.8 | 427.1 | 13.29 | 405.5 | 12.12 | 458.1* | 9.87 | 425.6 | 9.36 |
| Fe (mg) | 8.2 | 0.19 | 7.9† | 0.18 | 7.9* | 0.19 | 7.4 | 0.16 | 8.0* | 0.13 | 7.6 | 0.12 |
| Zn (mg) | 5.9† | 0.14 | 5.6† | 0.14 | 5.3 | 0.11 | 5.0 | 0.11 | 5.5* | 0.08 | 5.2 | 0.09 |
| Vit A (RAE) | 623.5* | 34.02 | 507.1† | 17.76 | 574.0* | 39.2 | 443.4 | 19.02 | 593.3* | 26.1 | 467.6 | 13.03 |
| Vit B1 (mg) | 0.7† | 0.03 | 0.7† | 0.02 | 0.6 | 0.02 | 0.6 | 0.02 | 0.7 | 0.02 | 0.7 | 0.01 |
| Vit B2 (mg) | 1.0 | 0.03 | 0.9 | 0.02 | 1.0 | 0.03 | 0.9 | 0.02 | 1.0* | 0.02 | 0.9 | 0.02 |
| Vit B12 (ug) | 3.0 | 0.1 | 3.0† | 0.11 | 3.1* | 0.18 | 2.6 | 0.09 | 3.1* | 0.11 | 2.7 | 0.07 |
| Vit C (mg) | 36.6† | 1.68 | 40.2† | 1.94 | 28.7 | 1.73 | 24.6 | 1.3 | 31.8 | 1.21 | 30.6 | 1.14 |
| Vit D (ug) | 4.7† | 0.22 | 4.1† | 0.18 | 3.1 | 0.16 | 3.1 | 0.17 | 3.7 | 0.13 | 3.5 | 0.12 |

* Mean value was at the highest and signiﬁcantly different between the sexes (P<0.05).

† Mean value was at the highest and signiﬁcantly different between the urban and rural areas (P<0.05).

**Supplementary Table 4. Percentage of children consuming selected macronutrients and micronutrients below the local EAR by age group, sex, and residence**

|  | **Urban** | | | **Rural** | | | **Urban and Rural** | | |
| --- | --- | --- | --- | --- | --- | --- | --- | --- | --- |
| **Parameter** | **Boys** | **Girls** | **Total** | **Boys** | **Girls** | **Total** | **Boys** | **Girls** | **Total** |
| **0.5 – 0.9 years** |  |  |  |  |  |  |  |  |  |
| Calcium | 1.9† | 3.6 | 2.8 | 0.3 | 3.4* | 1.6 | 1.0 | 3.5* | 2.1 |
| Iron | 65.3 | 71.1 | 68.3 | 59.0 | 67.2 | 62.2 | 61.5 | 69.2 | 65.0 |
| Zinc | 3.0† | 1.2 | 2.1 | 0.7 | 5.3* | 2.5 | 1.6 | 3.2 | 2.3 |
| Vitamin B1 | 53.6*† | 36.2 | 44.4 | 31.1 | 45.5 | 36.8 | 40.1 | 40.5 | 40.3 |
| Vitamin B2 | 28.9* | 22.1 | 25.3 | 19.3 | 28.4 | 22.9 | 23.1 | 25.1 | 24.0 |
| Vitamin B12 | 68.0† | 69.0 | 68.6† | 46.2 | 62.3 | 52.5 | 54.9 | 65.9 | 59.9 |
| Vitamin C | 34.3 | 39.2 | 36.9 | 38.7 | 45.2 | 41.3 | 37.0 | 42.0 | 39.3 |
| Vitamin A | 1.8 | 0.9 | 1.4 | 0.4* | 0.0 | 0.2 | 1.0 | 0.5 | 0.7 |
| **1 – 3.9 years** |  |  |  |  |  |  |  |  |  |
| Calcium | 44.5 | 61.9* | 52.9 | 67.6† | 65.3 | 66.4 | 59.4 | 64.1 | 61.7 |
| Iron | 8.0 | 8.5† | 8.2 | 11.6 | 15.4 | 13.5 | 10.3 | 13.0 | 11.6 |
| Zinc | 9.1 | 7.6† | 8.4 | 13.8 | 17.9 | 15.8† | 12.1 | 14.3 | 13.2 |
| Vitamin B1 | 24.6* | 26.4 | 25.5 | 36.3† | 32.9 | 34.6 | 32.2 | 30.7 | 31.4 |
| Vitamin B2 | 12.9 | 12.9† | 12.9 | 16.1 | 16.6 | 16.4 | 15.0 | 15.3 | 15.2 |
| Vitamin B12 | 20.2* | 16.6 | 18.4 | 32.0 | 26.9 | 29.5 | 27.8* | 23.4 | 25.6 |
| Vitamin C | 41.1 | 42.9 | 42.0 | 63.5 | 64.7 | 64.1 | 55.6 | 57.2 | 56.4 |
| Vitamin A | 14.3 | 26.2* | 20.1 | 30.3 | 28.2 | 29.3 | 24.7 | 27.5 | 26.0 |
| Vitamin D | 74.1 | 80.8* | 77.3 | 85.3 | 92.4 | 88.8 | 81.3 | 88.4* | 84.8 |
| **4 – 6.9 years** |  |  |  |  |  |  |  |  |  |
| Calcium | 73.1 | 87.4* | 78.2 | 83.5 | 84.9 | 84.1 | 78.7 | 85.9 | 81.6 |
| Iron | 23.5† | 14.2 | 20.2 | 7.2 | 11.5 | 9.1 | 14.7 | 12.5 | 13.9 |
| Zinc | 35.5 | 29.5 | 33.4 | 29.3 | 42.5 | 35.0 | 32.2 | 37.5 | 34.3 |
| Vitamin B1 | 29.0 | 31.5 | 29.9 | 31.9 | 28.9 | 30.6 | 30.5 | 29.9 | 30.3 |
| Vitamin B2 | 11.5 | 12.1 | 11.7 | 17.4 | 12.7 | 15.3 | 14.7 | 12.4 | 13.8 |
| Vitamin B12 | 16.1 | 14.9 | 15.7 | 17.7 | 17.2 | 17.5 | 17.0 | 16.3 | 16.7 |
| Vitamin C | 69.4 | 61.3 | 66.5 | 74.4 | 78.4 | 76.2 | 72.1 | 71.9 | 72.0 |
| Vitamin A | 40.7 | 57.2 | 46.6 | 38.4 | 37.4 | 38.0 | 39.5 | 45.0 | 41.7 |
| Vitamin D | 83.9 | 88.4 | 85.5 | 92.4* | 84.6 | 89.0 | 88.5 | 86.0 | 87.5 |
| **7-12.9 years** |  |  |  |  |  |  |  |  |  |
| Calcium | 95.2 | 92.3 | 93.6 | 95.9 | 97.9† | 97.0† | 95.6 | 95.8 | 95.7 |
| Iron | 23.8* | 22.8 | 23.2 | 24.4 | 29.6 | 27.3 | 24.1 | 27.0 | 25.8 |
| Zinc | 59.3 | 53.2 | 55.9 | 71.2 | 71.1† | 71.1† | 66.9 | 64.3 | 65.5 |
| Vitamin B1 | 73.1 | 63.9 | 67.9 | 86.4† | 78.7† | 82.2† | 81.6* | 73.1 | 76.8 |
| Vitamin B2 | 71.3* | 47.6 | 57.9 | 65.9 | 58.2 | 61.6 | 67.9* | 54.2 | 60.2 |
| Vitamin B12 | 48.8 | 46.7 | 47.6 | 60.8 | 59.5† | 60.0 | 56.4 | 54.6 | 55.4 |
| Vitamin C | 87.2 | 76.5 | 81.1 | 85.6 | 88.4† | 87.1 | 86.2 | 83.9 | 84.9 |
| Vitamin A | 68.6 | 49.1 | 57.6 | 76.4 | 78.9† | 77.8† | 73.6 | 67.7 | 70.3 |
| Vitamin D | 95.7* | 92.5 | 93.9 | 99.7*† | 99.5† | 99.6† | 98.2 | 96.8 | 97.5 |
| **0.5-12.9 years** |  |  |  |  |  |  |  |  |  |
| Calcium | 70.3 | 77.4* | 73.8 | 78.8† | 83.3 | 81.1 | 75.5 | 81.1* | 78.3 |
| Iron | 23.5 | 22.7 | 23.1 | 19.9 | 25.0† | 22.5 | 21.3 | 24.1 | 22.7 |
| Zinc | 36.9 | 35.3 | 36.1 | 42.1 | 50.3† | 46.3 | 40.1 | 44.6 | 42.4 |
| Vitamin B1 | 47.9 | 48.3 | 48.1 | 57.5 | 57.7 | 57.6 | 53.8 | 54.1 | 53.9 |
| Vitamin B2 | 37.4* | 32.2 | 34.8† | 39.1 | 39.1 | 39.1 | 38.4 | 36.5 | 37.5 |
| Vitamin B12 | 34.3 | 37.2* | 35.7† | 42.8† | 44.7 | 43.8 | 39.5 | 41.8 | 40.7 |
| Vitamin C | 67.7 | 63.7 | 65.8 | 74.1 | 78.9† | 76.5 | 71.6 | 73.2 | 72.4 |
| Vitamin A | 43.3 | 41.5 | 42.4 | 50.6 | 56.0 | 53.3 | 47.8 | 50.5 | 49.1 |
| Vitamin D | 86.8 | 89.1 | 88.0 | 94.0† | 95.1 | 94.6 | 91.2 | 92.9 | 92.0 |

* Prevalence (%) was at the highest and signiﬁcantly different between the sexes (P<0.05).

† Prevalence (%) was at the highest and signiﬁcantly different between the urban and rural areas (P<0.05).
